# Effects of chemical alternation on damage accumulation in concentrated solid-solution alloys

**DOI:** 10.1038/s41598-017-04541-8

**Published:** 2017-06-23

**Authors:** Mohammad W. Ullah, Haizhou Xue, Gihan Velisa, Ke Jin, Hongbin Bei, William J. Weber, Yanwen Zhang

**Affiliations:** 1 Materials Science and Technology Division, Oak Ridge National Laboratory, Oak Ridge, TN 37831 USA; 2 Department of Materials Science and Engineering, University of Tennessee, Knoxville, TN 37996 USA

## Abstract

Single-phase concentrated solid-solution alloys (SP-CSAs) have recently gained unprecedented attention due to their promising properties. To understand effects of alloying elements on irradiation-induced defect production, recombination and evolution, an integrated study of ion irradiation, ion beam analysis and atomistic simulations are carried out on a unique set of model crystals with increasing chemical complexity, from pure Ni to Ni_80_Fe_20_, Ni_50_Fe_50_, and Ni_80_Cr_20_ binaries, and to a more complex Ni_40_Fe_40_Cr_20_ alloy. Both experimental and simulation results suggest that the binary and ternary alloys exhibit higher radiation resistance than elemental Ni. The modeling work predicts that Ni_40_Fe_40_Cr_20_ has the best radiation tolerance, with the number of surviving Frenkel pairs being factors of 2.0 and 1.4 lower than pure Ni and the 80:20 binary alloys, respectively. While the reduced defect mobility in SP-CSAs is identified as a general mechanism leading to slower growth of large defect clusters, the effect of specific alloying elements on suppression of damage accumulation is clearly demonstrated. This work suggests that concentrated solid-solution provides an effective way to enhance radiation tolerance by creating elemental alternation at the atomic level. The demonstrated chemical effects on defect dynamics may inspire new design principles of radiation-tolerant structural alloys for advanced energy systems.

## Introduction

A new direction of research to substantially enhance alloy performance has been stimulated in material science due to the recent success in fabrication of single-phase concentrated solid-solution alloys (SP-CSAs)^1–5^. Historically, alloy development with desired performance focused on traditional alloys, where enhanced radiation resistance relied largely on unique microstructural heterogeneity to mitigate displacement damage^6^. In contrast to traditional alloys (containing one or two principle elements), SP-CSAs contain two to five or more elements at high concentration, sometimes at equal or near-equal concentration. As a result, the concept of “solvent” and “solute” in traditional alloys is irrelevant^6^. Although SP-CSAs contain 2–5 equal or near-equal concentration of elements, interestingly almost all SP-CSAs have either fcc or bcc structures. The formation of phases depends mainly on the enthalpy of mixing (Δ*H*_*mix*_), entropy of mixing (Δ*S*_*mix*_) and atomic size difference (*δ*)^3, 7^. According to Gibbs free energy of mixing Δ*G*_*mix*_ = Δ*H*_*mix*_ − *T*Δ*S*_*mix*_, entropy can stabilize a phase by lowering free energy with higher entropy, provided that enthalpy is constant.

In these CSAs, the random arrangement of multiple elemental species on a crystalline lattice results in atomic-level elemental alternation that creates disordered local chemical environments. Two distinctive intrinsic properties are expected: (1) unique electronic structures that significantly enhances scattering of electrons, reduces the thermal conductivity and affects energy dissipation; and (2) unique site-to-site lattice distortions that lead to complex energy landscapes and affect defect migration and mass transport. Compared to traditional alloys, SP-CSAs possess improved mechanical and chemical properties^7–10^, which may be attributed to the intrinsic properties of CSAs. The next generation nuclear power reactors will undergo harsh service conditions that include intense radiation flux, higher operating temperature, severe corrosive environment and high stress^11, 12^. SP-CSAs might be good candidate materials for nuclear power applications, if their superior mechanical properties can be complemented with high radiation resistance^13^. These alloys might also find applications in high-energy rare isotope beam facilities and concentrating solar power plants having extreme corrosive and radiation environments^14, 15^.

Austenitic steels are widely used as core structural material, as well as used for cladding on the inside surface of the pressure vessel of current water-cooled nuclear fission reactors. They are also proposed for next generation fission and fusion reactors, where excellent radiation resistance is the primary concern^12, 16^. Current austenitic steels are susceptible to void swelling, irradiation-assisted stress corrosion cracking, decrease in fracture toughness and radiation-induced segregation (RIS)^17, 18^. The RIS can provoke phase instability, embrittlement, and stress corrosion cracking issues^12, 19^, whereas void swelling can trigger unacceptable dimensional expansion and can lead to degradation of fracture toughness^18, 20^. Due to these adverse effects of radiation, austenitic steels are facing new challenges in the applications of next generation nuclear reactors. Recently, systematic ion beam study on FeNiMnCr SP-CSA has been peroformed at temperatures ranging from room temperature to 700 °C and midrange doses of 0.03–10 dpa using 5.8 MeV Ni ion. The FeNiMnCr alloy has demonestrated significant suppression of RIS, no observable void formation and lower defect cluster size compared to conventional single phase Fe-Cr-Ni and Fe-Cr-Mn austenitic alloys^21^. An enhancement of radiation tolerance with the suppression of void formation have been reported in Ni-based CSAs at elevated temperature with a peak damage dose of 60 dpa^5^.

Despite the recent intensive investigations on SP-CSAs, relatively few studies have been performed on their response to prolonged irradiation. Egami *et al*. have reported that, due to atomic level stresses and chemical heterogeneity, thin films of simple model binary alloys and Zr-Hf-Nb solid solution alloys show high radiation resistance under electron irradiation^13^. Molecular dynamics (MD) simulations of the cumulative irradiation of Fe-based binary alloys, having different percentages of Ni and Cr, show more damage accumulation in the face-centered cubic (fcc) alloys than in the body-centered cubic (bcc) ones^22^. Recently, MD simulations and experimental studies on irradiation of equiatomic single-phase NiFe and NiCoCr alloys have demonstrated noticeable damage reduction, as compared to pure Ni^23^. The observed damage reduction is explained by the reduced dislocation mobility, which results in smaller and fewer defect clusters in the alloys compared to elemental Ni. This result is supported by a recent experimental study on irradiation induced defect evolution in Ni and NiFe binary alloys; where at low-fluences, the equiatomic NiFe alloy demonstrated higher radiation resistance than pure Ni^24^.

In reactor environments, irradiation-induced point defect formation, migration and evolution are the primary reasons for the microstructural changes that affect the performance of structural materials. Controlling defect formation and migration in structural materials is, therefore, critical for designing materials with high radiation tolerance. While a few studies have demonstrated the potential benefit of improved irradiation resistance in SP-CSAs, especially the link between energy dissipation and defect evolution due to ferro- and anti-ferromagnetic interactions (the first distinctive aspect of these SP-CSAs)^25^, we utilize a set of model systems with increasing chemical complexity to reveal the second distinctive aspect of SP-CSAs by showing a unique link between modified energy landscapes and defect dynamic processes – an underappreciated but remarkable research field.

In this paper, we study the production and accumulation of point defects in pure Ni and Ni-based binary and ternary alloys. The binary and ternary alloys selected for this study are all single-phase concentrated solid solutions with various chemical complexity and yet have a simple fcc structure^26^. This model system includes pure Ni, Ni_80_Fe_20_ and Ni_50_Fe_50_ with increasing elemental randomness at the atomic level. The two binaries (Ni_80_Fe_20_ and Ni_80_Cr_20_) result from randomly replacing 20% of the atoms in Ni with either Fe or Cr, and the ternary Ni_40_Fe_40_Cr_20_ is formed by further replacing half of the Ni atoms in Ni_80_Cr_20_ with Fe atoms. Some of the model crystals of interest in this work (without the element Co that was included in some previous studies of stable concentrated solid-solution alloys^25–27^) are not far from a few of the alloys already under consideration for nuclear applications, but are based on different design principals^12, 28^. In this work, damage production and accumulation in these alloys due to irradiation are investigated using ion irradiation experiments and MD simulations. Although, the radiation dose (<0.13 dpa) of current work is considerably lower than the realistic applications, it demonstrates that the damage behavior of model SP-CSAs can be modified at the early stage in radiation environment. Our hypothesis is that modifying alloy chemical complexity (composition complexity) will enable us to control defect dynamics at the early stage of radiation damage to ultimately enhance radiation tolerance at the later stage under extreme radiation conditions^29^. The research focus is to: (1) understand the chemical effect of changing alloy compositions and elements but still retaining a simple fcc structure; and (2) demonstrate that controlled atomic-level elemental alternation in concentrated solid-solution alloys is an effective design principle to create modified energy landscapes that can have substantial impact on defect evolution. Such knowledge is important for new defect engineering paradigms benefiting broader science and technology.

## Results and Discussion

### Irradiation response in Ni and Ni-based alloys

Recent studies have suggested that compositional complexity strongly affects the electrical, thermal and magnetic properties of concentrated solid-solution alloys^25, 26^. Electrical resistivity exhibits a strong dependence on the type of elements, and the CSAs containing Cr exhibit an order of magnitude higher resistivity than those alloys without Cr. To evaluate alloying or concentration effects, the crystals chosen in this work have different percentages of Fe and Cr content. By investigating the irradiation response of model Ni and Ni alloys, we will gain insights on how modifying alloying elements and concentration affects defect dynamics.

**Figure 1:**
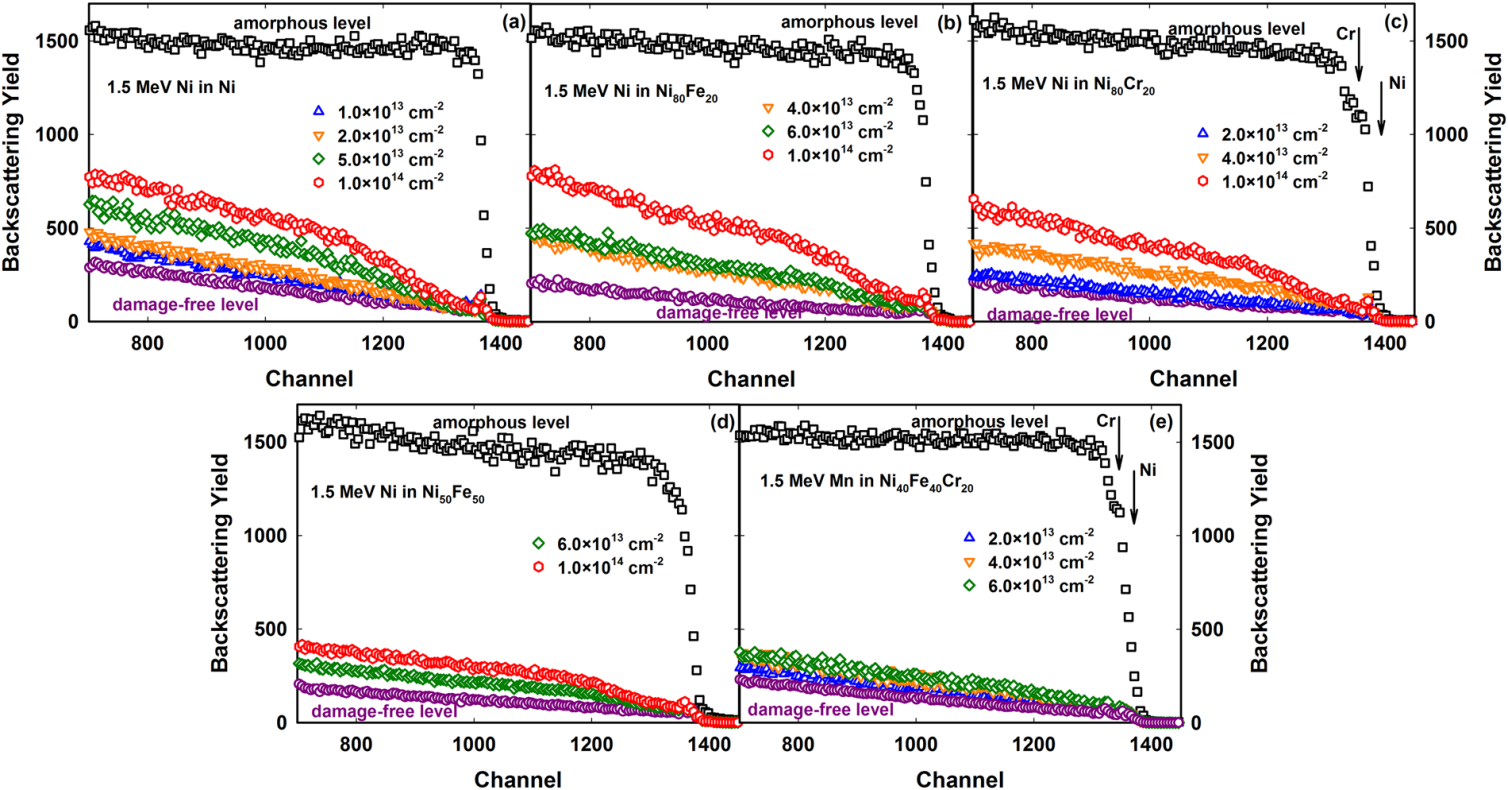
RBS spectra of the irradiated alloys. Spectra recorded from (**a**) Ni, (**b**) Ni_80_Fe_20_, (**c**) Ni_80_Cr_20_ and (**d**) Ni_50_Fe_50_ single crystals irradiated with 1.5 MeV Ni ions, and (**e**) Ni_40_Fe_40_Cr_20_ irradiated with 1.5 MeV Mn ions, at various ion fluences. Both virgin and random spectra are included to indicate the damage-free level before irradiation and the fully amorphous (or random) level, where no crystallinity exists. The higher backscattering yield suggests more irradiation-induced damage. For visual clarity, only every five data points are shown.

The RBS/C spectra for 1.5 MeV Ni ion irradiated elemental Ni, Ni_80_Fe_20_, Ni_80_Cr_20_, and Ni_50_Fe_50_ are shown in Fig. 1a–d, respectively, together with the spectra for Ni_40_Fe_40_Cr_20_ irradiated with 1.5 MeV Mn ions (Fig. 1e). A virgin spectrum along the <001> direction, which corresponds to pristine single crystals, and a random spectrum, which corresponds to a fully amorphous structure, are included. The *χ*_*min*_ for the virgin spectra is less than 6% that indicates the high crystalline quality for all the samples. The backscattering yield increases with increasing ion fluence, which suggests accumulation of radiation damage. The 80:20 binaries show better radiation resistance compared to elemental Ni at low ion fluences (<6.0 × 10^13^ cm^−2^), as indicated by the lower backscattering yields. When comparing the response of Ni_80_Fe_20_ and Ni_80_Cr_20_ to Ni_50_Fe_50_, a further enhancement of the radiation resistance is observed for Ni_50_Fe_50_. No significant difference in radiation response is identified under current irradiation conditions between the ferro- or anti-ferromagnetic couplings of Ni–Fe or Ni–Cr in Ni_80_Fe_20_ and Ni_80_Cr_20_. The clear damage suppression observed in Ni_50_Fe_50_ may be attributed to local environmental changes, such as the number and arrangement of the first and second neighbors of Ni or Fe with increasing Fe concentration from 20% to 50%. The results in Fig. 1 suggest that the intrinsic properties of elemental species and local arrangement (concentration) are key factors for controlling defect dynamics. In addition, the results from Fig. 1 do not indicate a significant difference in radiation resistance between the ternary Ni_40_Fe_40_Cr_20_ alloy and the binary Ni_50_Fe_50_ alloy beyond the uncertainty of the experimental measurements. These results emphasize that the concentration of specific elements could be as effective as the number of elements on tuning the irradiation response of CSAs.

### Damage accumulation

**Figure 2:**
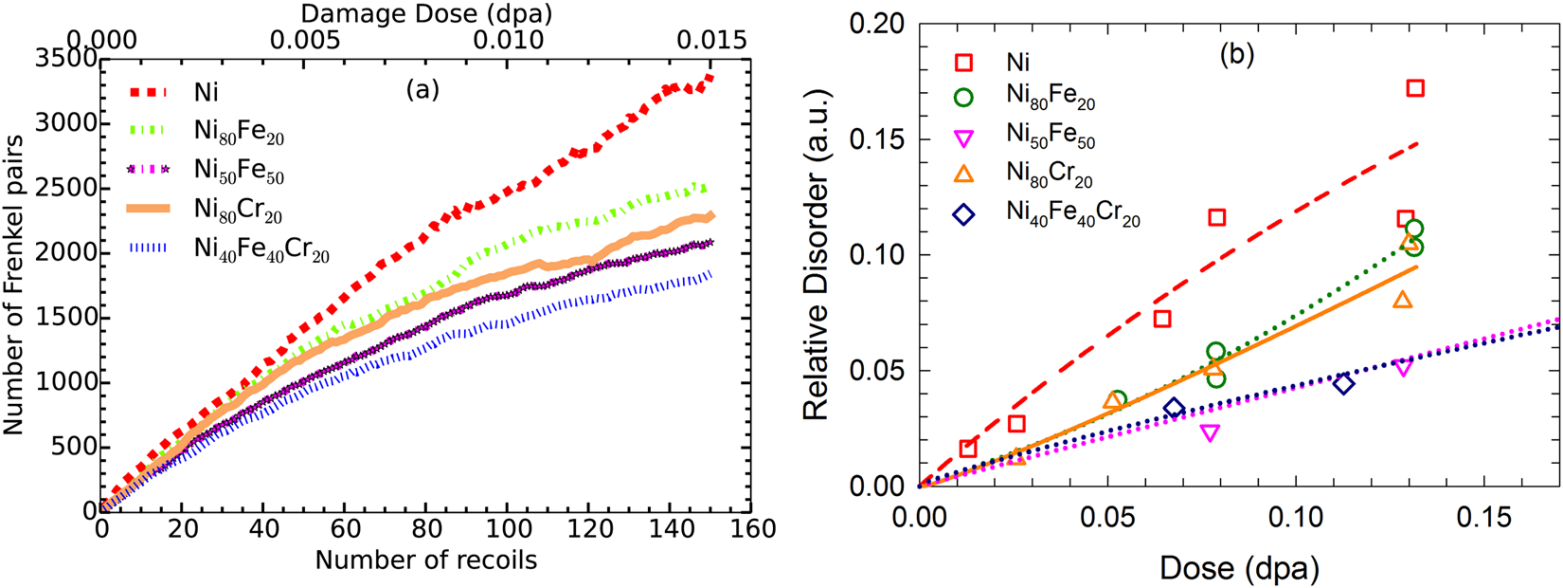
Comparison of damage accumulation. Damage accumulation in pure Ni, Ni_80_Fe_20_, Ni_50_Fe_50_, Ni_80_Cr_20_ and Ni_40_Fe_40_Cr_20_ alloys from both irradiation simulation and experiment. (**a**) Accumulation of point defects with increasing numbers of cumulative recoils and the corresponding dose in dpa. This is an average of two sets of cumulative recoil simulations. (**b**) Relative disorder in the model systems as the function of dose at damage peak (~400 nm).

In this section, we elaborate on the damage accumulation in the model alloys using both atomistic simulations and experiments. Increased radiation resistance of equiatomic NiCo and NiFe alloys compared to pure Ni has been reported previously under Au ion irradiation. The results showed that the damage yield in NiCo is about half that in Ni, while the damage yield in NiFe is even lower compared to NiCo^25^. To examine the role of increasing complexity from simple solid solutions to more complex, we compare damage production and accumulation under prolonged overlapping cascade events in the model crystals, going from simple Ni and binaries to more complex Ni_40_Fe_40_Cr_20_. From the MD simulations, the evolution of FPs with respect to the number of overlapping recoils in these model systems is given in Fig. 2a. The results show that the highest numbers of FPs are produced in pure Ni, compared to the other alloys. In the binary alloys, Ni_50_Fe_50_ exhibits fewer radiation-induced FPs than the 80:20 alloys. The difference in defect production between Ni_80_Fe_20_ and Ni_80_Cr_20_ is evident for the higher number of recoils (>90 recoils) or dose; whereas the numbers of FPs largely overlap at lower numbers of recoils (<50 recoils). A similar trend was previously observed for the accumulation of 5 keV recoil cascades, where Ni_80_Cr_20_ produced 1.7 times fewer FPs than the Ni_80_Fe_20_ alloy^30^. Compared to 5 keV cascades, the difference in radiation tolerance between these two alloys is less pronounced for 25 keV recoils.

The ternary Ni_40_Fe_40_Cr_20_ alloy exhibits the best radiation resistance to the accumulation of 25 keV recoil cascades, producing the lowest number of FPs compared to the binaries and pure Ni. The damage accumulation curves are nearly linear and coincident with each other for pure Ni and the binary 80:20 alloys at low doses (below 50 recoils); whereas in case of Ni_40_Fe_40_Cr_20_, the defect accumulation curve starts to deviate from linearity and from the other alloys at a much lower dose, corresponding to about 10 recoil cascades. The total number of FPs produced in the Ni_40_Fe_40_Cr_20_ alloy is approximately a factor of 2.0 lower compared to pure Ni and approximately 1.4 times lower than the 80:20 binary alloys. This behavior suggests very efficient in-cascade damage recombination and dynamic annealing in the ternary alloy compared to pure Ni and the binary alloys. In the conventional MD simulations, due to limited time scale, the dose rate is several orders of magnitude higher than for a reactor environment or for the ion irradiation experiments shown in Fig. 1 and Fig. 2b. Under actual experimental conditions, the point defects have longer time to migrate and recombine between cascade events. As a result, the actual damage accumulation rate in the MD simulations may be overestimated, but the relative difference or trend in defect production, cluster formation and damage accumulation behavior should be valid using this approach.

By employing an iterative process that is described in detail in ref. 31, the relative disorder profiles are derived from the RBS/C spectra and plotted as a function of dose (in dpa) in Fig. 2b for Ni irradiated Ni, Ni_80_Fe_20_, Ni_50_Fe_50_, Ni_80_Cr_20_ and Mn irradiated Ni_40_Fe_40_Cr_20_, respectively. A disorder level of “0” refers to a pristine single crystal, and a level of “1” corresponds to a fully amorphous phase. Some data are taken from a previous publication^32^. At relatively low doses (up to ~0.13 dpa), enhanced radiation resistance is confirmed as the elemental complexity increases from Ni to the ternary alloy. On the other hand, the damage evolution of Ni_50_Fe_50_ and Ni_40_Fe_40_Cr_20_ are similar, indicating the need for understanding different coupling strengths between elements.

Based on both the simulation (Fig. 2a) and experimental results (Fig. 2b) presented above, we can divide the radiation response of these materials into three groups. In the first group, pure Ni is very sensitive to ion irradiation. In the second group, the 80:20 binary alloys moderately suppress damage accumulation, as compared to Ni. Finally, Ni_50_Fe_50_ and Ni_40_Fe_40_Cr_20_ alloys demonstrate the best radiation response based on their damage accumulation behavior. By increasing randomness in the NiFe alloy from 80:20 to 50:50, the damage accumulation is suppressed very efficiently, and the damage level is comparable to that of the ternary alloy at lower dose (below 50 recoils). There is good qualitative agreement between simulation and experiment, particularly at lower doses. In both cases, we see overlapping damage levels at the lowest doses; whereas the damage profiles start to deviate from each other at higher doses, following the three groups discussed above.

The results in Figs 1 and 2 demonstrate how material properties can be altered dramatically by varying the elemental type and concentration of alloying elements. A previous study demonstrated that Ni_33_Co_33_Cr_33_ and Ni_25_Co_25_Fe_25_Cr_25_ outperformed Ni_50_Fe_50_ in radiation resistance; whereas Ni_33_Fe_33_Co_33_ and Ni_50_Fe_50_ are comparable^6^. This evidence, including the results shown in Figs 1 and 2, suggest strong coupling of Cr‒Co and Ni‒Fe, but relatively weak coupling of Ni‒Cr in the Ni-based alloys, may be used to tune the atomic transport and defect dynamics in a non-equilibrium radiation environment. This hypothesis also suggests that the radiation response of alloys is not simply dependent on the number of alloying elements, but is also affected by the coupling of specific elements.

### Damage in single cascade

**Figure 3:**
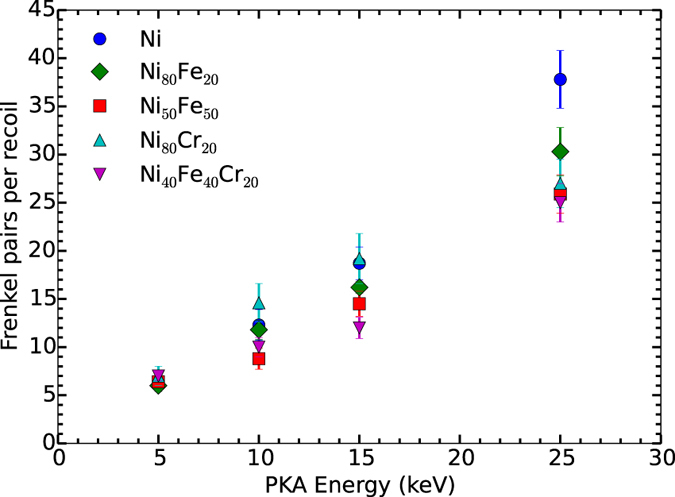
Comparison of number of Frenkel pairs. Number of Frenkel pairs produced in a single impact of 5, 10, 15 and 25 keV cascade energies. Data are taken as an average of ten simulations. Error bars show the standard error of the mean.

In comparison to previous results for 5 keV primary knock-on atom (PKA) cascades^30^, the average numbers of FPs produced in a single cascade as a function of PKA energy for these compositions are shown in Fig. 3. The results show a non-linear trend of defect production with increasing energy of recoil. The difference in the number of FPs between the alloys for all PKA energies is within the limits of uncertainty, except for pure Ni. On the other hand, we see a clear difference of defect production in overlapping cascade simulations for both 5 keV^30^ and 25 keV (Fig. 2) damage accumulation curves. This indicates that in-cascade damage recombination during irradiation plays an important role in the suppression of defect accumulation in these alloys. There is no noticeable difference between Ni and Ni-based alloys in defect production for 5 keV single cascades. In contrast, we see significant damage reduction in alloys compared to pure Ni for 25 keV single recoils.

### Interstitial cluster formation and evolution

**Figure 4:**
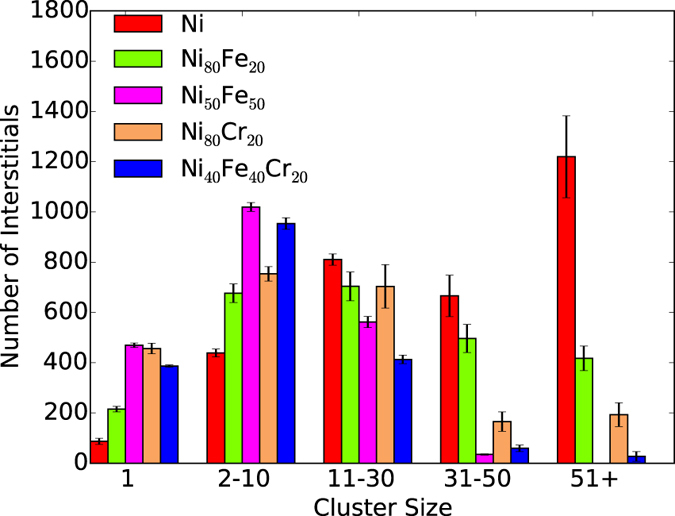
Interstitial cluster size distribution. Comparison of interstitial cluster size distribution between Ni, Ni_80_Fe_20_, Ni_50_Fe_50_, Ni_80_Cr_20_ and Ni_40_Fe_40_Cr_20_ alloys after 150 cumulative recoils (0.015 NRT dpa). Data are taken as an average of two sets of recoil simulations. Error bars show the standard error of the mean.

In the previous section, overall defect production and accumulation are shown to vary significantly in different alloys. In this section, we focus on the clustering of point defects in the alloys under irradiation. Figure 4 compares the interstitial clustering behavior for all the alloys after 150 overlaps of 25 keV cascades. In pure Ni, interstitials primarily form medium to large defect clusters, with a number of clusters exceeding 50 interstitials in size, and only a few isolated interstitials (cluster size of 1). On the other hand, small and medium size defect clusters containing 2 to 30 point interstitials are the main features of the binary alloys. There are some additional differences in the size distribution of the interstitial clusters in the Ni_80_Fe_20_ and Ni_80_Cr_20_ alloys. The number of interstitials in large clusters (cluster size of 31–50 and 51+) is relatively higher in Ni_80_Fe_20_, compared to Ni_80_Cr_20_. A different cluster formation behavior is observed for Ni_50_Fe_50_ and Ni_40_Fe_40_Cr_20_ alloys, which tends more toward a smaller cluster size. In other words, the cluster size distribution for these alloys is quite the opposite of that found in pure Ni. In Ni_50_Fe_50_ and Ni_40_Fe_40_Cr_20_ alloys, large cluster formation is negligible (cluster size of 31–50 and 51+); instead, interstitials primarily remain as clusters containing 2–10 point defects or as isolated point defects. In all compositions, medium and large interstitial clusters often produce dislocation loops lying on the {111} plane.

**Figure 5:**
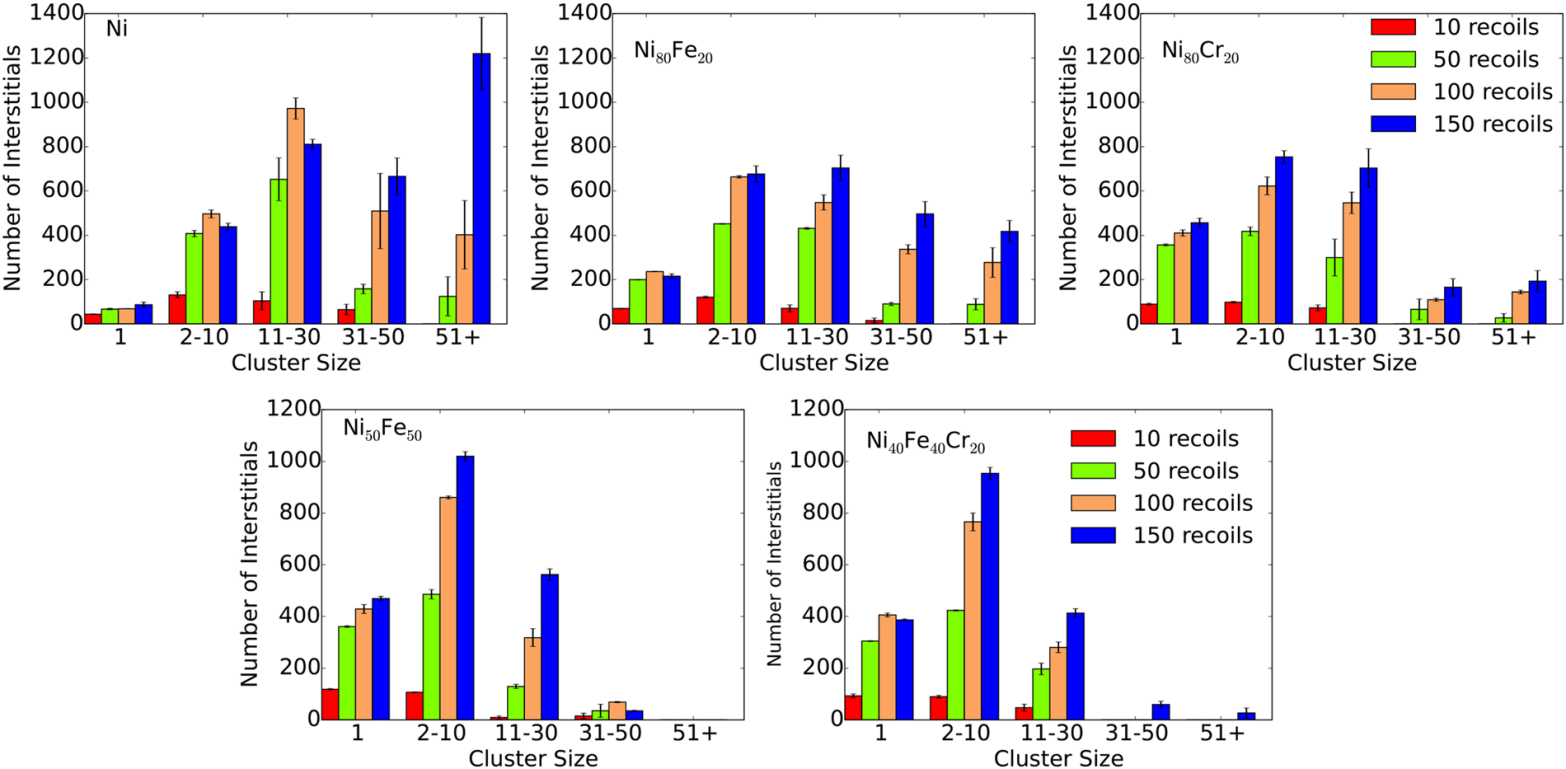
Interstitial cluster size distribution at different recoil events. Comparison of interstitial cluster size distribution between Ni, Ni_80_Fe_20_, Ni_50_Fe_50_, Ni_80_Cr_20_ and Ni_40_Fe_40_Cr_20_ alloys at 10, 50, 100 and 150 recoil events. Data are taken as an average of two sets of recoil simulations. Error bars show the standard error of the mean.

To understand the growth mechanism of the final interstitial clusters presented in Fig. 4, we turn our focus to the evolution of interstitial cluster sizes at different number of recoils. Figure 5 shows the interstitial cluster size distribution with increasing recoil number for pure Ni and the alloys. The newly created interstitials at higher recoil numbers demonstrate a preference to diffuse to a specific cluster size based on alloy composition. This preference can be divided into three categories. First, in pure Ni, new point defects primarily contribute to the growth of big defect clusters with increasing number of recoils; whereas single point defects saturate at 10 overlapping cascades. This is evident from the growth rate of the 51+ defect clusters compared to other cluster sizes. Second, in the 80:20 binary alloys, cluster sizes between 2 and 30 dominate and increase with the number recoils; whereas isolated interstitials saturate at 50 overlapping cascades. In particular, the number of isolated interstitials decreases slightly in the Ni_80_Fe_20_ alloy (150 recoils), which suggests that the rate of recombination and diffusion of isolated defects are higher than for newly created interstitials. Finally, in Ni_50_Fe_50_ and Ni_40_Fe_40_Cr_20_ alloys, new defects mainly contribute to the growth of small clusters (2–10 defects), and isolated point defects saturate at around 100 overlapping cascades, particularly for the ternary alloy.

The cluster analysis results shown in Fig. 5 suggest that with increasing cascade overlap, some point defects are effectively annealed due to recombination; at the same time, some interstitials diffuse to grow clusters and form dislocation loops. Such cluster size variation is attributed to alloy composition or chemical complexity. The cluster formation behavior can be explained by taking into account the mobility of interstitials among the different compositions. Interstitial mobility and diffusivity is highest in pure Ni compared to the alloys. Aidhy *at el*. have reported that interstitials are more mobile in pure Ni than the Ni_50_Fe_50_ and Ni_80_Cr_20_ alloys^33^. Defect formation and migration energy in Ni and Ni-based binary and ternary alloys are studied using *ab initio* calculation based on density functional theory, classical MD and kinetic activation-relaxation technique (k-ART)^34–36^. Vacancy move through the Fe sublattice, whereas Ni-Ni dumbbells are the most stable form of interstitial due to lower formation energy and they prefer to move through Ni sublatttice. In alloys, amount of Ni sublattice is reduced compared to pure Ni, this means slow diffusion of interstitials. For example, in pure Ni, the interstitial atom can jump to any of the 12 nearest neighbours, whereas the jump number will be limited in alloys. There are non-favorable atom pairs, even in a small cluster of interstitials. The migration energy of interstitial is also higher in alloys compared to pure Ni, which also contributes to the slow diffusion of interstitials. The migration energy of vacancy in Ni and Ni-based alloys is very high and very low diffusivity at low temperature. So, at room temperature and in MD time scale damage evolution is mainly contributed by interstitial diffusion. It has also been shown that the diffusivity of Ni atoms in pure Ni is higher than in the Ni_45_Fe_40_Cr_15_ ternary alloy^37^. Diffusion is even more sluggish in the five component (CoCrFeMnNi) high-entropy alloys^38^. Due to high mobility and diffusivity, isolated point defects can interact with each other and more readily form defect clusters in Ni. With increasing cascade overlap, large clusters form at the expense of small clusters and isolated point defects. In the binary and ternary alloys, small and medium sized clusters dominate due to the restricted mobility of point defects and small clusters. Significant suppression of radiation induced void swelling is reported in Ni-based SP-CSAs at 500 °C. Enhanced swelling resistance is attributed to the tailored migration behavior of interstitial defect clusters^5^. Very recently, Granberg and coworkers have studied edge dislocation mobility in pure Ni compared with NiFe and NiCoCr alloys^23^. They have concluded that the dislocations are less mobile in NiFe as compared to pure Ni, and the ternary NiCoCr alloy shows even lower mobility than the binary NiFe. Thus, compared to pure Ni, small clusters in these alloys cannot diffuse easily to form larger clusters. Dislocation mobility is lowest in the third category of alloys, namely Ni_50_Fe_50_ and Ni_40_Fe_40_Cr_20_, which results in only one cluster consisting of more than 50 interstitials in Ni_40_Fe_40_Cr_20_ and no large clusters in the Ni_50_Fe_50_ alloy (Fig. 4).

### Vacancy cluster formation and evolution

**Figure 6:**
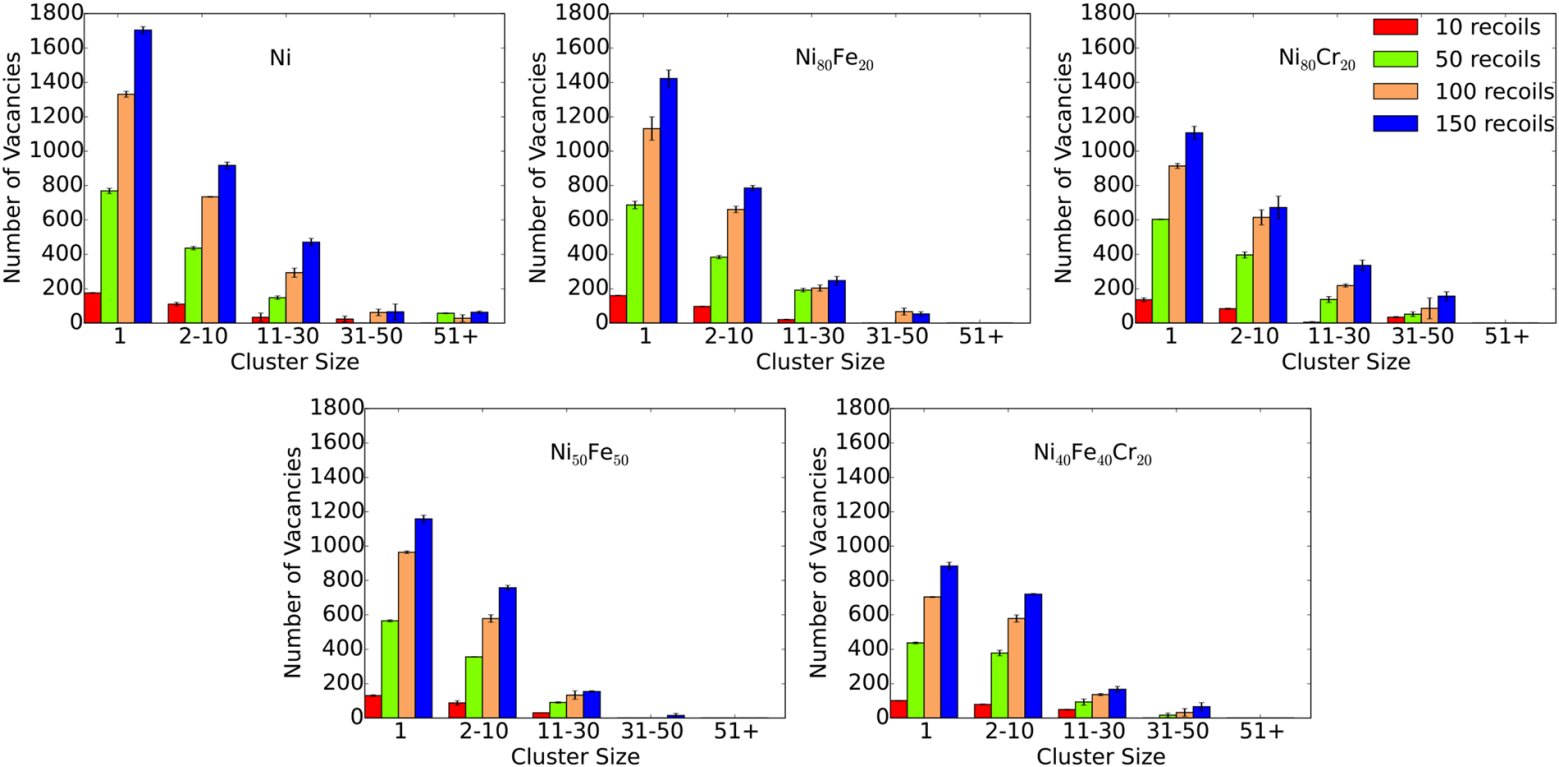
Vacancy cluster size distribution at different recoil events. Comparison of vacancy cluster size distribution between Ni, Ni_80_Fe_20_, Ni_50_Fe_50_, Ni_80_Cr_20_ and Ni_40_Fe_40_Cr_20_ alloys at 10, 50, 100 and 150 recoil events. Data are taken as an average of two sets of recoil simulations. Error bars show the standard error of the mean.

The numbers of vacancies in different cluster size distributions are summarized in Fig. 6. Cluster formation dynamics is very different for vacancies than for interstitials. Vacancies typically remain as isolated point defects and small clusters (2–10 point defects) for all the materials studied. The number of vacancies forming clusters decreases with increasing cluster size. Binary and ternary alloys do not produce any large vacancy clusters (>51 vacancies). The number of vacancies in medium sized clusters (31–50 vacancies) are also insignificant compared to isolated vacancies. We have previously reported a similar pattern of vacancy cluster size distribution for the accumulation of 5 keV recoil cascades^30^. Studies have shown that vacancies are immobile until 700 K, and start to diffuse around 1000 K in pure Ni^33^. The probability of isolated vacancies and small clusters to diffuse and form larger vacancy clusters is therefore low. In general, Ni produces relatively larger vacancy clusters compared to other alloys, due to both a higher vacancy yield in Ni and a lower vacancy mobility in the alloys. Small vacancy cluster formation in pure Ni and the alloys (as shown in Fig. 6) might therefore result from a violent collision process and short-time (a few ps) cascade heating rather than from long-term diffusion processes (ns or longer).

**Figure 7:**
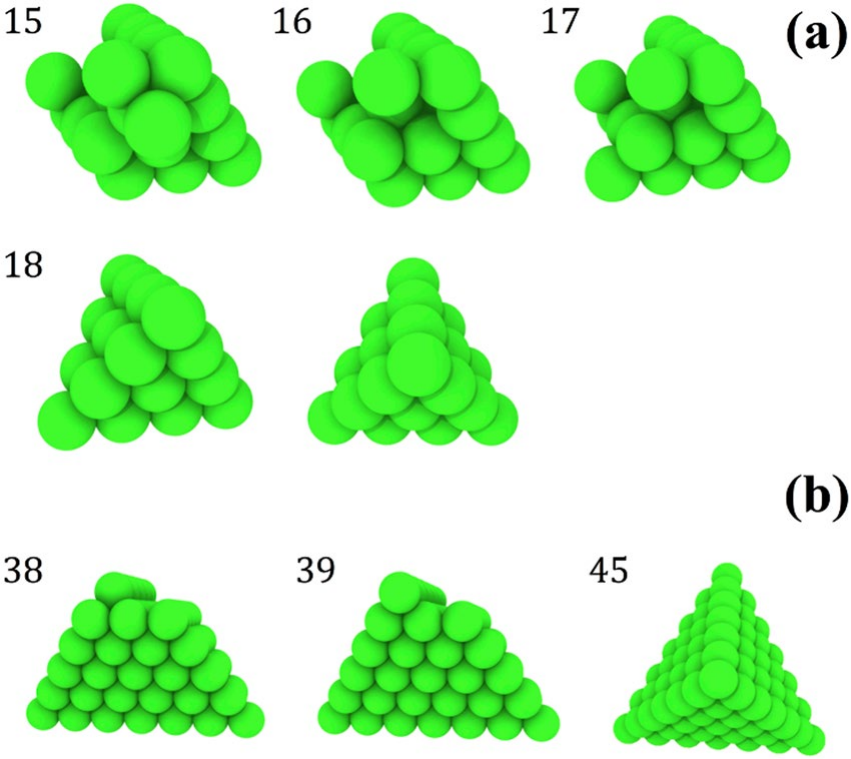
SFT formation. Illustration of the growth of a perfect stacking fault tetrahedron as a function of recoil number, where spheres represent empty lattice sites (vacancies). (**a**) A perfect stacking fault tetrahedron formation in Ni_80_Fe_20_, gradually formed at 15, 16, 17 and 18 consecutive recoils. The final view shows a different angle of the SFT after the 18^th^ recoil. (**b**) Gradual SFT formation in Ni at 38, 39 and 45 recoils.

Figure 7 shows the evolution of vacancy clusters to form SFT in Ni_80_Fe_20_ and elemental Ni. After the 15^th^ recoil in Ni_80_Fe_20_ (Fig. 7a), a vacancy cluster is formed with one well-defined {111}-side plane. No vacancy cluster was found to exist for fewer recoils, so this cluster formed entirely during the 15^th^ recoil event. After some rearrangement of vacancies following the 16^th^ and 17^th^ consecutive recoils, a perfect SFT with an edge length of four vacancies forms during the 18^th^ recoil. Figure 7b presents irradiation-assisted formation of similar vacancy-like SFT in Ni with an edge length of seven vacancies. Other binary and ternary alloys produce truncated SFT. Besides single isolated SFT, some coupling of SFT along an edge length or a plane are also observed. It is important to note that only a small fraction of defects produce perfect SFT. Other defects consist of isolated point defects, defect clusters and truncated SFT. A few studies have been carried out to understand the formation mechanism of SFT in fcc metals during ion irradiation by MD simulation^39, 40^. In particular, Nordlund *et al*. have shown that, in fcc Cu and Au, SFT can form directly in the atom-depleted zone at the center of a collision cascade by four intersecting {111} vacancy stacking faults and simultaneous atom rearrangement^39^. The SFT observed in the current study can also be described using this method due to the simulation conditions and features of the SFT. Experimentally, SFT are observed in high purity (containing Fe, Ni and Cr) and model austenitic alloys, irradiated at both high and low doses^41, 42^. In contrast, SFT are either absent or present in high density in commercial austenitic alloys, depending on the impurity content^43, 44^.

Previous high resolution transmission electron microscopy (HRTEM) studies of irradiated Ni and Ni-based alloys have revealed both interstitial type dislocation loops and vacancy-type SFT. Lu *et al*. have observed SFT in single crystalline pure Ni, NiCo and NiFe alloys irradiated with 3 MeV Au ions to a fluence of 5 × 10^15^/cm^2^ ^27^. The vacancy-type SFT are mostly dispersed in the matrix but there are some coupled SFT that form parallelograms. Interstitial dislocation loops of about 1–8 nm diameter have also been observed under TEM^33^. A previous study of comparison between MD simulation and TEM image suggests that higher number of large defect clusters are produced in elemental Ni compared to binary and ternary alloys^23^. Therefore, the irradiation-induced defect structures of current simulations are in good agreement with experimental observations.

## Conclusions

Damage accumulation due to cumulative irradiation of Ni, Ni_80_Fe_20_, Ni_80_Cr_20_, Ni_50_Fe_50_ and Ni_40_Fe_40_Cr_20_ alloys has been studied in both ion irradiation experiments and molecular dynamics simulations. The single crystals of these alloys have been irradiated with 1.5 MeV Ni or Mn ions at fluences ranging from 1 × 10^13^ cm^−2^ to 1 × 10^14^ cm^−2^. In MD simulations, overlapping cascades of 25 keV recoils have been used to simulate damage accumulation. The binary and ternary alloys show efficient suppression of defect production and accumulation. The accumulated damage is not linear with either increasing number of recoils or recoil energy, indicating the importance of in-cascade damage recombination in the final state of damage production. Larger interstitial clusters are produced in Ni; whereas, the average cluster size decreases when 20% of the Ni atoms in pure Ni are substituted with Fe or Cr. Even further reduction of damage accumulation is evident in Ni_50_Fe_50_ and Ni_40_Fe_40_Cr_20_, both of which exhibit increasing randomness of local atomic arrangements that significantly modify the first and second nearest neighbors. The results indicate a decrease in defect mobility with increasing alloy complexity, which is also supported by previous experimental and simulation studies. SFT-like vacancy clusters are produced in all compositions. The results from using both experimental irradiations and MD simulations in these integrated studies suggest that changing the complexity of concentrated solid solution alloys can alter defect accumulation and evolution under extended irradiation environments. Strong coupling of Cr‒Co and Ni‒Fe, but relatively weak coupling of Ni‒Cr, in the Ni-based concentrated solid solution alloys may be utilized to tune the atomic transport and defect dynamics in a non-equilibrium radiation environment. This combination emphasizes that the radiation response of complex alloys is not simply dependent on the number of alloying elements but is also affected by the coupling of specific elements. This approach of designing materials based on manipulation of alloy complexity may result in new radiation tolerant materials.

## Methods

### Experiment

The ion irradiation and ion beam analysis of single crystal Ni, Ni_80_Fe_20_, Ni_50_Fe_50_, Ni_80_Cr_20_ and Ni_40_Fe_40_Cr_20_ were performed at the Ion Beam Materials Laboratory (IBML)^45^ at the University of Tennessee, Knoxville. 1.5 MeV Ni (for Ni, Ni_80_Fe_20_, Ni_50_Fe_50_ Ni_80_Cr_20_) and 1.5 MeV Mn (for Ni_40_Fe_40_Cr_20_) ion irradiations were performed to produce damage profiles with peak values of disorder at a depth of around 400 nm in order to minimize surface sink effects^46^. During ion irradiation, a defocused ion beam was wobbled over a 3.0 × 3.0 cm^2^ area by two raster scanners at 517 Hz (horizontal) and 64 Hz (vertical) to ensure uniformity^45^. An ion flux of 3.5 × 10^11^ cm^−2^ was used to reach ion fluence ranged from 1.0 × 10^13^ cm^−2^ to 1.0 × 10^14^ cm^−2^. The corresponding doses were predicted using the stopping and range of ions in matter (SRIM) code, under the quick calculation of damage option, and ranged from 0.01 to 0.13 dpa (displacements per atom)^47^. A displacement threshold energy of 40 eV was applied for Ni, Fe and Cr in all SRIM calculations. After ion irradiations, Rutherford backscattering spectrometry in channeling geometry (RBS/C) using 3.5 MeV He ions was performed *in-situ* along the <001> direction. All the ion irradiations and RBS/C measurements were performed at room temperature under a high vacuum^25, 45^.

Atom probe tomography (APT) analyses on the Ni-xFe (x = 0–60) alloys have shown that these alloys remain random solid solution after the Ni ion irradiations at room temperature to a dose of ~6.5 dpa, which is beyond the dose range of the present study^32^. Moreover, recent neutron diffraction studies^48^ have revealed only fcc peaks for the Ni_80_Cr_20_ alloys at room temperature without superlattice peaks (e.g. Ni_2_Cr), confirming a single-phase solid solution. Furthermore, our own microstructure and X-ray diffraction characterization of the Ni_40_Fe_40_Cr_20_ used here confirms a single-phase solid solution. Further studies are needed to investigate the phase stability of Ni_80_Cr_20_ and Ni_40_Fe_40_Cr_20_ under ion irradiation at room temperature to high doses, but we assume that these two alloys remain a random solid solution form at the low irradiation doses (<0.13 dpa) in the present study. The room temperature specific heat of these alloys are very similar from 0.4 to 0.5 J. g^−1^. K^−1^ but their values varies significantly with temperature due to magnetic state and possible short-range order-disorder transitions^48, 49^. The room temperature lattic parameters determinted using neutron diffraction measurement is largest for Ni, which is followed by Ni_80_Cr_20_ and Ni_50_Fe_50_. Below the Curie temperature, the thermal diffusivity and conductivity of Ni_50_Fe_50_ is higher than Ni_80_Cr_20_ but smaller than elemental Ni.

### Cascade simulation

The recoil simulations were performed using the LAMMPS and PARCAS codes, which are based on classical MD methods^50–52^. The LAMMPS code was used to create a random mixture of elements to mimic the random nature of SP-CSAs. Irradiation simulations were performed using the MD code PARCAS. In the simulations, a variable time step is used, where the time step is inversely proportional to the total force that the recoil atom experiences and its velocity, using a proportionality constant^50^. The interaction between atoms in pure Ni, Ni_50_Fe_50_, Ni_80_Fe_20_, Ni_80_Cr_20_ and Ni_40_Fe_40_Cr_20_ alloys were described using the embedded atom method (EAM) potential by Bonny *et al*.^53^. This potential was developed to study the formation and evolution of radiation defects, particularly point defects produced during collision cascades. This is suitable to model stable fcc NiFeCr compositions with a focus on compositions around the Fe_70_Ni_10_Cr_20_ alloy, and not suitable for the study of phase stabilities. Besides the bcc phase, this potential does not consider phases like FeCr sigma, Ni_2_Cr and FeNi_2_. The self-diffusion coefficients of different constituent elements in the NiFeCr alloys determined using this potential are in the order *D*_*cr*_ > *D*_*Ni*_ > *D*_*Fe*_. The self-diffusion coefficient of Cr is higher, which is in good agreement with broad experimental observations. In the case of Ni and Fe, both *D*_*Ni*_ > *D*_*Fe*_ and *D*_*Fe*_ > *D*_*Ni*_ observations are reported^54–56^.

The simulation cell size was 284 × 284 × 284 Å^3^ containing 2,050,000 atoms. Initially, the cell was relaxed at 300 K and zero pressure, utilizing a Berendsen temperature and pressure control^57^. Periodic boundary conditions were applied in all directions to replicate an infinitely large bulk system^58^. The electronic stopping was applied as a friction force for atoms with kinetic energies higher than 1 eV and calculated using the SRIM code version 2013^47, 50^. The effects of damage accumulation in Ni, Ni_80_Fe_20_ and Ni_80_Cr_20_ alloys due to the overlapping of 5 keV recoil cascades have been studied previously^30^. In this paper, 25 keV recoil cascades were employed to account for the higher energies in the recoil spectra from ion and neutron irradiation, and the results are compared to some previous results for 5 keV recoil cascades and with experiment. To mimic the random nature of overlapping cascades, the simulation cell was shifted by a random displacement vector between zero and the cell size in the [100], [010] and [001] directions before each subsequent recoil simulation. After that, one recoil was initiated by a Ni atom close to the center of the simulation cell with an energy of 25 keV in a random direction. This allowed a uniform distribution of crystal damage and avoided cascades interacting with the cell borders. The simulation time (100 ps) was chosen so that the system cooled down and no energetic interactions remained in the system. Details of the simulation procedure can be found elsewhere^22, 30^. A total of 150 recoil simulations were performed to achieve a damage dose equivalent to 0.015 NRT-dpa, which yielded good statistics for characterization of the final damage state^59^.

### Defect analysis of the simulation cells

The Voronoi cell method was used to identify vacancies and interstitials. In this approach, atoms of the defective crystal configuration were assigned to Wigner-Seitz-cell-Voronoi polyhedra centered at the lattice sites of the ideal reference crystal. Polyhedra with no atoms were labeled as vacancies and polyhedra with 2 or more atoms were labeled as interstitials^51^.

In addition, a cluster connectivity analysis was done for all investigated cases. Interstitial and vacancy clusters were identified separately. Defects identified by the Voronoi cell method were used as input for this analysis. All defects within a fixed cut-off radius from each other were interpreted to be a part of the same defect cluster. The cut-off radius was set as the second-nearest-neighbor distance (3.65 Å), which is the general cut-off distance used in several previous studies^22, 60^. Defects were visualized with the program OVITO^61^.

The Voronoi cell method is reliable for determining the number of surviving Frenkel pairs (FPs), but it cannot identify the structures with correlated atomic displacements typical of stacking faults. To identify stacking fault tetrahedra (SFT), the equivalent sphere (ES) method of defect analysis was used. A sphere radius of 0.3a_0_, where a_0_ is the lattice parameter, was used to reliably detect SFT. Details of the analysis method can be found in other publications^22, 39, 62^.
